# The Effect of PRMT1-Mediated Arginine Methylation on the Subcellular Localization, Stress Granules, and Detergent-Insoluble Aggregates of FUS/TLS

**DOI:** 10.1371/journal.pone.0049267

**Published:** 2012-11-13

**Authors:** Atsushi Yamaguchi, Keiko Kitajo

**Affiliations:** Department of Neurobiology, Graduate School of Medicine, Chiba University, Chiba, Japan; Hertie Institute for Clinical Brain Research and German Center for Neurodegenerative Diseases, Germany

## Abstract

*Fused in sarcoma/translocated in liposarcoma (FUS/TLS*) is one of causative genes for familial amyotrophic lateral sclerosis (ALS). In order to identify binding partners for FUS/TLS, we performed a yeast two-hybrid screening and found that protein arginine methyltransferase 1 (PRMT1) is one of binding partners primarily in the nucleus. *In vitro* and *in vivo* methylation assays showed that FUS/TLS could be methylated by PRMT1. The modulation of arginine methylation levels by a general methyltransferase inhibitor or conditional over-expression of PRMT1 altered slightly the nucleus-cytoplasmic ratio of FUS/TLS in cell fractionation assays. Although co-localized primarily in the nucleus in normal condition, FUS/TLS and PRMT1 were partially recruited to the cytoplasmic granules under oxidative stress, which were merged with stress granules (SGs) markers in SH-SY5Y cell. C-terminal truncated form of FUS/TLS (FUS-dC), which lacks C-terminal nuclear localization signal (NLS), formed cytoplasmic inclusions like ALS-linked FUS mutants and was partially co-localized with PRMT1. Furthermore, conditional over-expression of PRMT1 reduced the FUS-dC-mediated SGs formation and the detergent-insoluble aggregates in HEK293 cells. These findings indicate that PRMT1-mediated arginine methylation could be implicated in the nucleus-cytoplasmic shuttling of FUS/TLS and in the SGs formation and the detergent-insoluble inclusions of ALS-linked FUS/TLS mutants.

## Introduction

Amyotrophic Lateral Sclerosis (ALS) is the most frequent form of adult-onset fatal progressive motor neuron diseases, characterized by the degeneration of upper and lower motor neurons. Approximately ∼10% of ALS patients are familial [1]. Recently various mutations in the genes coding for two DNA/RNA binding proteins, TAR DNA binding protein-43 (TDP-43) and fused in sarcoma/translocated in liposarcoma (FUS/TLS), were identified in familial ALS cases [2], [3], [4], suggesting the molecular mechanisms regulating RNA metabolism could be implicated in familial ALS pathogenesis [1], [5]. On the other hand, the presence of abnormal protein aggregates in neuron or glia is one of key characteristics for most neurodegenerative disorders. Postmortem analysis in ALS patients showed abnormal cytoplasmic aggregations of these DNA/RNA binding proteins in affected neurons [1], [2], [3], [4], [6]. These results indicate that gain of toxic function and/or loss of function of these DNA/RNA binding proteins might be implicated in the pathogenesis of ALS [6], [7], [8]. Additionally mutations in *TDP-43* and *FUS/TLS* gene were identified in the cases of frontotemporal lobar degeneration (FLTD), and ubiquitin-positive inclusions in some cases of FTLD contain these protein products as a major component [2], [9], [10], [11], [12], supporting that ALS and FTLD might be a part of a clinical, pathologic, and genetic disease spectrum.

FUS/TLS, originally identified as a component of fusion oncogenes in human cancers, is a ubiquitously expressed 526 amino acids protein that belongs to the FET/TET family of multifunctional DNA/RNA binding proteins, including Ewing’s sarcoma protein and the TATA-binding protein associated factor TAF15 [13]. FUS/TLS, continuously nucleo-cytoplasm shuttling, contains an N-terminal Gln-Gly-Ser-Tyr (QGSY)-rich region, a Gly (G)-rich region, an RNA recognition motif (RRM), two Arg-Gly-Gly (RGG) repeats divided by a zinc finger motif, and a highly conserved extreme C-terminus that encodes a non-classic nuclear localization signal (NLS) recognized by transportin [14]. FUS/TLS is involved in mRNA splicing [15], DNA repair [16], pairing of homologous DNA and cell proliferation [17], transcriptional regulation [18], and the transport of mRNA for local translation in neuronal dendrites [19]. The vast majority of ALS-linked mutations are clustered in the C terminal NLS, most of which result in the retention and the inclusion of FUS/TLS mutants in the cytoplasm [1], [5].

Arginine methylation is catalyzed by protein-arginine-*N-*methyltransferases (PRMTs) utilizing S-adenosyl-L-methionine (AdoMet) as the donor of methyl group. PRMT1, a member of 11 PRMTs family, is a type I methyltransferase that transfers a methyl group from *AdoMet* to guanidino nitrogens of arginine residues to form mono-methyl and asymmetric dimethyl arginine [20], [21]. PRMT1 is the major asymmetric arginine methyltransferase, contributing to as much as 85% of all cellular PRMT activity. PRMT1, located both in the nucleus and in the cytoplasm, is highly mobile between these compartments. Since there are enzymes that likely remove this modification, arginine methylation is not a static post-translational modification, indicating that arginine methylation could be a rapid modification of protein functions [21], [22].

Stress granules are dense RNP-containing cytoplasmic bodies that arise during cell stress (heat, hypoxia, oxidative conditions, viral infection, and ultraviolet irradiation). The core constituents of SGs are translationally silent 48S pre-initiation complex, early initiation factors (eIF3, eIF4), and RNA-binding proteins (TIA-1, TIAR) [23], [24]. Stress granules could serve as a component of an adaptive mechanism sequestering and protecting cytoplasmic mRNAs in stress conditions [25]. TDP43 and FUS/TLS are recruited to SGs under stress conditions such as heat and oxidative stress [26], [27], [28], [29], [30], indicating these DNA/RNA binding proteins could be implicated in mRNA metabolism in stress conditions.

In the present study, we performed a yeast two-hybrid screening on a human fetal brain cDNA library to identify interacting partners for FUS/TLS and found that PRMT1 is one of binding partners for FUS/TLS.

## Materials and Methods

### Cell Culture

Human embryonic kidney HEK293 and human neuroblastoma SH-SY5Y cells were cultured in Dulbecco’s modified Eagle’s medium (DMEM) supplemented with 10% fetal bovine serum (FBS) at 37°C in a 5% CO_2_ atmosphere. Transient transfections were performed using Lipofectamine 2000 (Invitrogen, CA, USA) according to the manufacturer’s instructions. In some cases, the cells were treated with 20 µM adenosine-2′,3′-dialdehyde (AdOx) (Sigma-Aldrich, #A7154), or with 0.5 mM sodium arsenite (Sigma-Aldrich, #35000) for 30 min and harvested as indicated. The effectiveness of AdOx treatment was optimized by treating the cells with various concentrations of AdOx (0–50 µM) and optimal concentration 20 µM was determined. Cell viability assay by trypan blue staining had no effect on the viability of the cells at this concentration.

### Yeast Two-hybrid Screening

Yeast two-hybrid screening was conducted using Matchmaker GAL4 two-hybrid system 3 (Clontech, CA, USA) as described previously [31]. The region containing full-length FUS/TLS (amino acid 1–526) was generated by PCR, subcloned downstream of the GAL4 DNA-binding domain in pGBKT7 (pGBKT7-FUS/bait), and introduced into the yeast strain AH109 as bait. The human fetal brain cDNA library in pGADT7 (Clontech) was introduced into the yeast strain Y187. These two yeast strains were combined according to the yeast mating protocol recommended by the manufacturer. Then the positive clones were further evaluated by X-α-Gal assay, in which colonies positive for α-Galactosidase turn blue on medium with 20 µg/ml X-α-Gal. The plasmids were isolated, and the sequencings were performed.

### Western Blot Analysis, Immunocytochemistry and Immunohistochemistry

Western blot analysis and immunohistochemistry were performed as described previously [32]. Cell extracts or brain homogenates for western blot analysis were prepared by lysing them in lysis buffer [50 mM Tris-HCl (pH 8.0), 20 mM EDTA, 1% NP-40, 100 mM NaCl, 10 mM *β* **-** *glycerophosphate,* protease inhibitor cocktail (Roche Diagnostics)]. The samples (20 µg/lane) were boiled in loading buffer [100 mM Tris-HCl (pH 6.8), 200 mM dithiothreitol, 4% SDS, 0.2% bromophenol blue, 0.2% glycerol] for 5 minutes, and subjected to electrophoresis on 10% SDS-PAGE. After the proteins were transferred onto polyvinylidene difluoride (PVDF) membrane (Millipore Corp.), the membrane was incubated in blocking buffer [phosphate-buffered saline (PBS) containing 0.05% Tween 20 (PBS-T) with 5% nonfat dried milk] for 1 hour at room temperature and then probed with a primary antibody in blocking buffer overnight at 4°C. The membrane was washed three times in PBS-T, probed with the secondary horseradish peroxidase-linked anti-mouse or -rabbit IgG antibody (Cell Signaling Biotechnologies) in blocking buffer for 1 hour at room temperature, and washed again in PBS-T. Detection of signal was performed with ECL chemiluminescence system (GE Healthcare Ltd).

Cells were fixed with 4% paraformaldehyde (PFA) for 15 minutes and then washed by PBS three times. Cells were permeabilized and non-specific sites were blocked by incubating with blocking solution [PBS containing 0.1% Triton X-100, 5% bovine serum albumin (BSA) and 3% goat serum]. The cells were incubated with primary antibodies diluted in a blocking solution overnight at 4°C. Then, the cells were washed in PBS and incubated with Alexa488 and Alexa568 (Invitrogen) conjugated secondary antibodies for 1 hour at room temperature. Then samples were stained with DAPI (4′,6-diamino-2-phenylindole) and mounted with a fluorescent mounting medium (DakoCytomation). Images were obtained using fluorescence microscopy (Nikon, E600).

The animal care and use protocol was approved by the Chiba University Animal Care and Use Committee. Adult mice were intracardially perfused with 4% PFA. Brains and lumbar spinal cord were harvested and post-fixed in 4% PFA overnight at 4°C, followed by sucrose replacement. Brains were embedded in Optimal Cutting Temperature (OCT) compound on a metal block in liquid nitrogen, and stored at −80°C. Frozen sections were cut in 30 µm-thick sections with a microtome. Antigen retrieval was performed by microwave-heated incubation in citrate buffer (10 mM Citric Acid, pH 6.0) for 20 minutes, followed by incubation with antibodies diluted in a blocking solution overnight at 4°C. Then, samples were washed in PBS and incubated with Alexa488 and Alexa568 (Invitrogen) conjugated secondary antibodies for 1 hour at room temperature. Samples were mounted with a fluorescent mounting medium (DakoCytomation). Images were obtained using fluorescence microscopy (Nikon, E600). The primary antibodies used were anti-FUS/TLS (A300-302A)(Bethyl Laboratories, Inc., TX, USA), anti-FUS/TLS (4H11)(Santa Cruz Biotechnologies, sc-47711), anti-PRMT1(A33) (Cell Signaling Technologies, #2449), anti-Histone H4 (F-9) (Santa Cruz Biotechnologies, sc-25260), anti-eIF3η (N-20) (Santa Cruz Biotechnologies, sc-16377), anti-TIAR (C-18) (Santa Cruz Biotechnologies, sc-1749), anti-HA (Sigma-Aldrich, HA-7), anti-c-Myc (Santa Cruz Biotechnology, 9E10), anti- GST (B-14) (Santa Cruz Biotechnologies, sc-138), anti-Neurofilament (Sigma-Aldrich, #N4142) and anti-NeuN (Millipore, #MAB377), anti-Actin (I-19) (Santa Cruz Biotechnologies, sc-1616) and anti-alpha-Tubulin (B-7) antibody (Santa Cruz Biotechnologies, sc-5286).

### Immunoprecipitation Assay

For immunoprecipitation, brain lysates (∼800 µg) or cell lysates (∼500 µg) were incubated with 1 µg of the indicated antibody in TNE lysis buffer [50 mM Tris-HCl (pH 8.0), 20 mM EDTA, 1% NP-40, 100 mM NaCl, protease inhibitor cocktail] in 2 ml eppendorf tubes. Protein-G coupled Sepharose beads (30 µl of a 50% slurry) were added into each tube. The beads were spun down, and the supernatant was removed. The beads were then washed three times with TNE lysis buffer. They were then resuspended in loading buffer, and boiled for 5 minutes. The supernatant was collected and analyzed by immunoblot.

### Cell Fractionation Assay

Preparation of cytosolic and nuclear fractions was performed as described previously [33]. Briefly, harvested cells from 80–90% confluent culture dish (∼1×10^7^ cells) were suspended in 100 µl Buffer A (10 mM HEPES, pH 7.9, 10 mM KCl, 0.1 mM EDTA, 0.1 mM EGTA, 0.15% NP-40) containing protease inhibitor and followed by 15 minutes incubation in 2 ml eppendorf tube. Homogenates were centrifuged at 12 000 *g* for 1 minute at 4°C, and the supernatant (cytosolic fraction) was stored at –80°C. The pellet was washed with 1 ml Buffer A, then resuspended in 100 µl Buffer B (20 mM HEPES, pH 7.9, 0.4 mM NaCl, 1 mM EDTA, 1 mM EGTA, 0.5% NP-40) containing protease inhibitor and sonicated at 4°C. Cellular debris was removed by centrifugation at 12 000 *g* for 30 minutes at 4°C and the supernatant (nuclear fraction) was stored at –80°C.

### Constructs and Mutagenesis

Human cDNAs for *FUS/TLS* and *PRMT1* transcript variant 3 were generated by PCR using HEK293 cDNA as template. *PRMT1* inactive mutant (*VLD63AAA*) was generated using the QuickChange site-directed mutagenesis kit (Stratagene, CA, USA). Sequences of the primers used are 5′-AAGGACAAGGTGGCAGCAGCCGTCGGCTCGGGCACCGGCA-3′ and 5′-TGCCGGTGCCCGAGCCGACGGCTGCTGCCACCTTGTCCTT-3′. cDNA for truncated *FUS-dC* (C-terminal NLS deletion mutant), encoding 1-513 aa of FUS/TLS, was generated by PCR using primers 5′-ATGGCCTCAAACGATTATACCCA-3′ and 5′- GGAATCCATCTTGCCAGGGC -3′. The resulting PCR products were subcloned into FPC1-HA, FPC1-Myc [34] or pcDNA4 expression vector (Invitrogen).

### Small-interfering RNA (siRNA) Assay

siRNA targeting PRMT1(h) (sc-41069) was purchased from Santa Cruz Biotechnologies. A non-targeting control siRNA (Santa Cruz Biotechnologies, sc-37007) was used as a negative control. Transfection of siRNA at 40 nM of final concentration was performed by *Lipofectamine*™2000 Transfection Reagent (Invitrogen) according to the manufacture’s protocol. Analysis was performed after 48 hours incubation.

### Tetracycline-inducible Expression *System*

Tetracycline-inducible PRMT1 over-expressing cells were established by T-Rex System (Invitrogen) according to the manufacturer’s protocol as described previously [32]. Briefly, N-terminal Myc-tagged human PRMT1 wild type or inactive form cDNA was subcloned into pcDNA4/TO plasmid (pcDNA4/TO-Myc-PRMT1). HEK293 cells were co-transfected with pcDNA4/TO-Myc-PRMT1 wt or inactive form and pcDNA6/TR, and then selected by cultivation in 100 µg/ml blastcidine S and 100 µg/ml Zeocin (Invitrogen). Inducible over-expression was confirmed by Western blot with anti-Myc antibody using cell lysates in the presence (Tet +) or absence (Tet −) of tetracycline (1 µg/ml) for 24 hours.

### *In vitro* and *in vivo* Methylation Assay

*In vitro* and *in vivo* methylation assay were performed as described previously with some modifications [33]. For *in vitro* methylation assay, recombinant His-MBP-tagged PRMT1 protein was obtained from ATGen Co. Ltd (Gyeonggi-do, Korea). Full-length FUS/TLS cDNA was subcloned into the pGEX-5X (GE Healthcare) bacterial expression vector to produce glutathione *S*-transferase (GST) fusion proteins for assay. GST fused-FUS/TLS (GST-FUS) or GST alone (control) was produced and purified as described by the manufacturer. Then 1 µg of recombinant PRMT1 (10 units) was combined with 1 µg GST-FUS or GST alone and 80 µM *S*-adenosyl- L-methionine (SAM)(New England Biolabs, #B9003S) in reaction buffer [20 mM Tris–HCl (pH 8.0), 200 mM NaCl, 0.4 mM EDTA], and reactions were incubated for 1 hour at 37°C. The reaction mixture was resuspended in loading buffer, boiled, resolved by SDS-PAGE, and transferred onto PVDF membrane. The methylation status was measured by Western blot with anti-Dimethyl Arginine (7E6) antibody (Novus Biologicals, #NB500-120) or Anti-dimethyl-Arginine Antibody, asymmetric (ASYM24, Millipore #07-414).

For *in vivo* methylation assay, FUS/TLS protein was obtained by the immunoprecipitation with anti-FUS/TLS antibody in lysates of HEK293 cells, and then resolved by SDS-PAGE. The methylation levels were measured by anti-Dimethyl Arginine (7E6) antibody or ASYM24.

### Extraction of Detergent-Insoluble Protein

To isolate Triton-insoluble fraction, cells were processed as previously described [35] with modifications. Briefly, cells were directly lysed in lysis buffer (2% Triton X-100, 150 mM KCl, protease inhibitor cocktail), sonicated, and shaken for 1 hour at 4°C. Samples were then centrifuged at 18,000×g for 30 minutes. The pellets were boiled in boiling buffer (2% SDS, 50 mM Tris HCl pH 6.8), and protein concentrations were quantified by DC protein assay (Bio-Rad, CA, USA).

### Quantification for Western Blot Bands

Western blot images were analysed with ImageJ software (National Institutes of Health), which evaluates the relative amount of protein staining with normalization to the corresponding controls.

### Statistical Analysis

Quantitative data were analyzed by the Student’s *t*-test, with *p*-values less than 0.05 considered as statistically significant. Data were expressed as means ± S.D. as indicated.

## Results

### PRMT1 is a Binding Partner for FUS/TLS Primarily in the Nucleus

To search for novel binding partners of FUS/TLS in the central nervous system, we performed a yeast two-hybrid screening on human fetal brain cDNA library using full length human FUS/TLS (amino acid 1-526) as a bait and identified several positive clones. Among them, at least six clones (#2, #9,#15, #56, #59 and #60) were identical to human *PRMT1* transcript variant 3 (accession number NM_198318). PRMT1, alternatively spliced to at least three variants, is a major protein arginine methyltransferase in mammalian cells [21], [22], [36] and this PMRT1 variant 3 used in the present study is shorter isoform compared to variant 1 or variant 2. The antibody against PRMT1, used in the present study, can detect three splicing isoforms of PRMT1.

**Figure 1:**
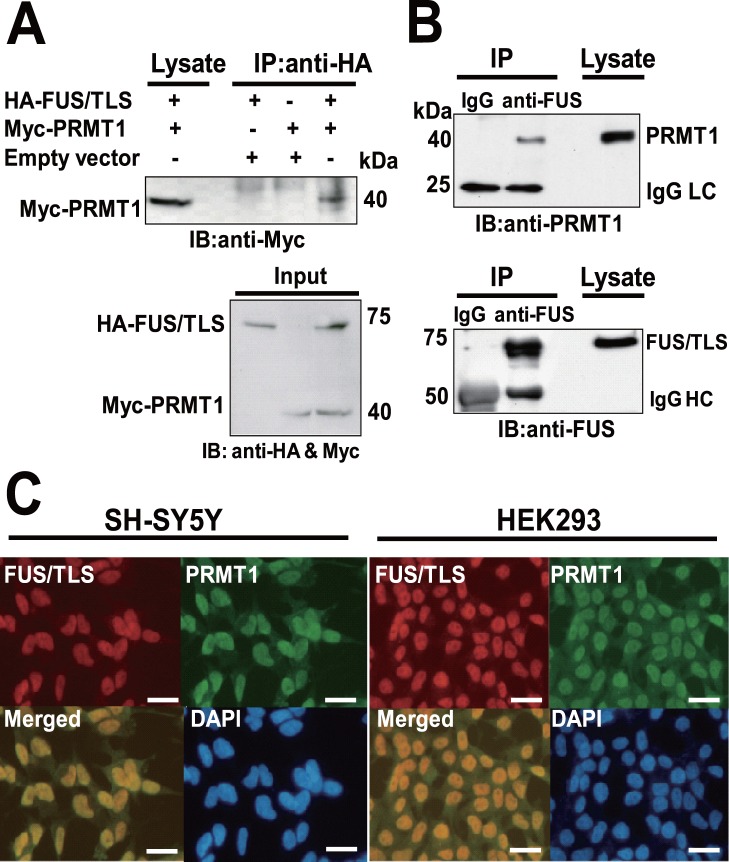
Interaction between FUS/TLS and PRMT1 in mammalian cells. (A) Co-immunoprecipitation assay between HA-tagged FUS/TLS and Myc-tagged PRMT1 protein. HEK293 cells were transiently transfected with the indicated plasmids. Cell lysates were immunoprecipitated with anti-HA antibody, and then immunoprecipitates were immunoblotted with anti-Myc antibody (upper panel). Cell lysates were immunoblotted with anti-HA and anti-Myc antibody (lower panel). IB, Immunoblot; IP, Immunoprecipitation. (B) Co-immunoprecipitation assay between endogenous FUS/TLS and PRMT1 protein. The lysates of HEK293 cells were immunoprecipitated with anti-FUS/TLS antibody or control mouse IgG. Then immunoprecipitates were immunoblotted with anti-PRMT1 (upper panel) or anti-FUS/TLS antibody (lower panel). IgG HC, Immunoglobulin heavy chain. IgG LC, Immunoglobulin light chain. (C) HEK293 and SH-SY5Y cells were co-stained with anti-FUS/TLS and anti-PRMT1 antibody, and followed by the incubation with Alexa488 and Alexa568 conjugated secondary antibody (Invitrogen). Samples were then stained with DAPI. Images were obtained using fluorescence microscopy (Nikon, E600). Bars, 20 µm.

To confirm the interaction between FUS/TLS and PRMT1 in mammalian cells, we performed a co-immunoprecipitation assay using HEK293 cells. HEK293 cells were transiently co-transfected with expression vectors encoding HA-tagged FUS/TLS (HA-FUS/TLS) and Myc-tagged PRMT1 (Myc-PRMT1) individually (with empty vector) or in a combination. Cell lysates were immunoprecipitated with anti-HA antibody, followed by the immunoblot with anti-Myc antibody (Fig. 1A, upper panel). The band for Myc-PRMT1 was detected in the immunoprecipitates with anti-HA antibody. The expression of HA-FUS/TLS and Myc-PRMT1 protein were confirmed by Western blot analysis using anti-HA and anti-Myc antibody (Fig. 1A, lower panel).

Next, to confirm the interaction between endogenous FUS/TLS and PRMT1, we performed a co-immunoprecipitation assay in HEK293 cells expressing FUS/TLS and PRMT1 endogenously. HEK293 cell lysates were immunoprecipitated with anti-FUS/TLS antibody or control IgG, followed by the immunoblot with anti-PRMT1 (Fig. 1B, upper panel) or anti-FUS/TLS antibody (Fig. 1B, lower panel). The band for PRMT1 was detected in the immunoprecipitates with anti-FUS/TLS antibody, but not in those with control IgG (Fig. 1B, upper panel), indicating the interaction between endogenous FUS/TLS and PRMT1. In addition, the band for FUS/TLS was detected in the immunoprecipitates with anti-FUS/TLS antibody, but not in those with control IgG (Fig. 1B, lower panel), indicating FUS/TLS protein was immunoprecipitated by anti-FUS/TLS antibody in this assay. Since PRMT1 binds to several RNA-binding proteins [21], [22], it is possible that the observed binding between FUS/TLS and PRMT1 was mediated by nucleic acids rather than direct interaction. To clarify whether FUS/TLS and PRMT1 are in an RNA/DNA-dependent complex, we performed an immunoprecipitation assay in the presence of nucleases (RNAase A and DNAase I) (Fig. S1) and found that FUS/TLS interacts with PRMT1 in the presence of nucleases, indicating FUS/TLS could directly interact with PRMT1.

We then examined the subcellular localization of endogenous FUS/TLS and PRMT1 in HEK293 and neuroblastoma SH-SY5Y cells by immunofluorescence assay (Fig. 1C). The immunoreactivities for FUS/TLS were primarily localized in the nucleus, and those for PRMT1 were also localized primarily in the nucleus with diffused cytoplasmic weak signals, indicating FUS/TLS and PRMT1 were co-localized primarily in the nucleus in HEK293 and SH-SY5Y cells.

### Interaction between FUS/TLS and PRMT1 in Mouse Brain and Spinal Cord

**Figure 2:**
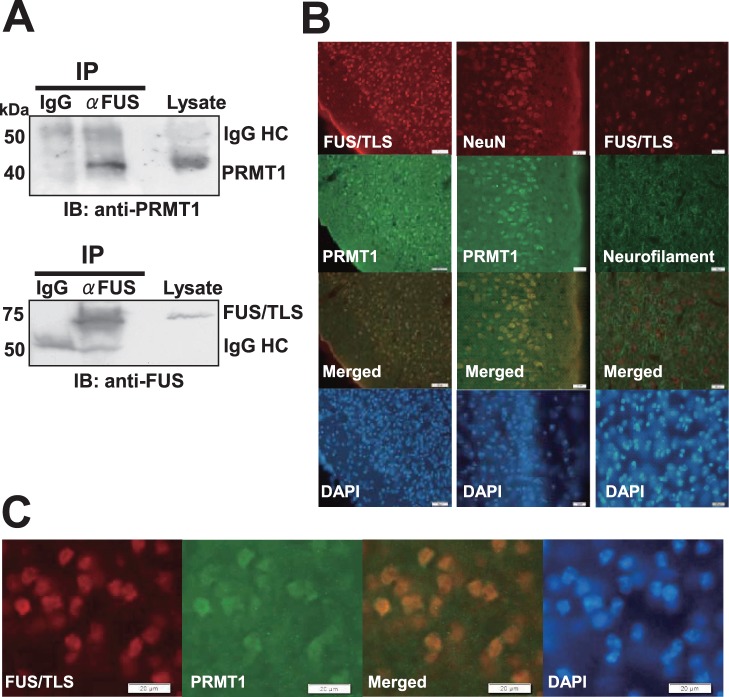
Interaction between FUS/TLS and PRMT1 in mouse brain cortex. (A) Co-immunoprecipitation assay between FUS/TLS and PRMT1 protein in mouse brain. Brain lysates were immunoprecipitated with anti-FUS/TLS antibody or control mouse IgG. Then immunoprecipitates were immunoblotted with anti-PRMT1 (upper panel) or anti-FUS/TLS antibody (lower panel). IgG HC, Immunoglobulin heavy chain. (B) Representative microphotographs (Objective lens, ×20) for the immunohistochemistry on formalin fixed mouse brain sections. Brain sections were co-stained with indicated antibodies and followed by the incubation with fluorescence conjugated secondary antibody. The samples were stained with DAPI and images were obtained using fluorescence microscopy. (C) Representative microphotographs at higher magnification (Objective lens, ×40) for the immunohistochemistry with indicated antibodies on formalin fixed mouse brain sections. The samples were stained with DAPI, and images were obtained using fluorescence microscopy.

To confirm the interaction between FUS/TLS and PRMT1 in the central nervous system (CNS), we performed a co-immunoprecipitation assay using mouse brain lysates. Adult mouse brain lysates were immunoprecipitated with anti-FUS/TLS antibody or control IgG, followed by the immunoblot with anti-PRMT1 (Fig. 2A, upper panel) or anti-FUL/TLS antibody (Fig. 2A, lower panel). The band for PRMT1 was detected in the immunoprecipitates with anti-FUS/TLS antibody, but not in those with control IgG (Fig. 2A, upper panel), indicating the interaction between endogenous FUS/TLS and PRMT1 in mouse brain. In addition, the band for FUS/TLS was detected in the immunoprecipitates with anti-FUS/TLS antibody, but not in those with control IgG (Fig. 2A, lower panel), indicating FUS/TLS protein was immunoprecipitated by anti-FUS/TLS antibody in this assay.

**Figure 3:**
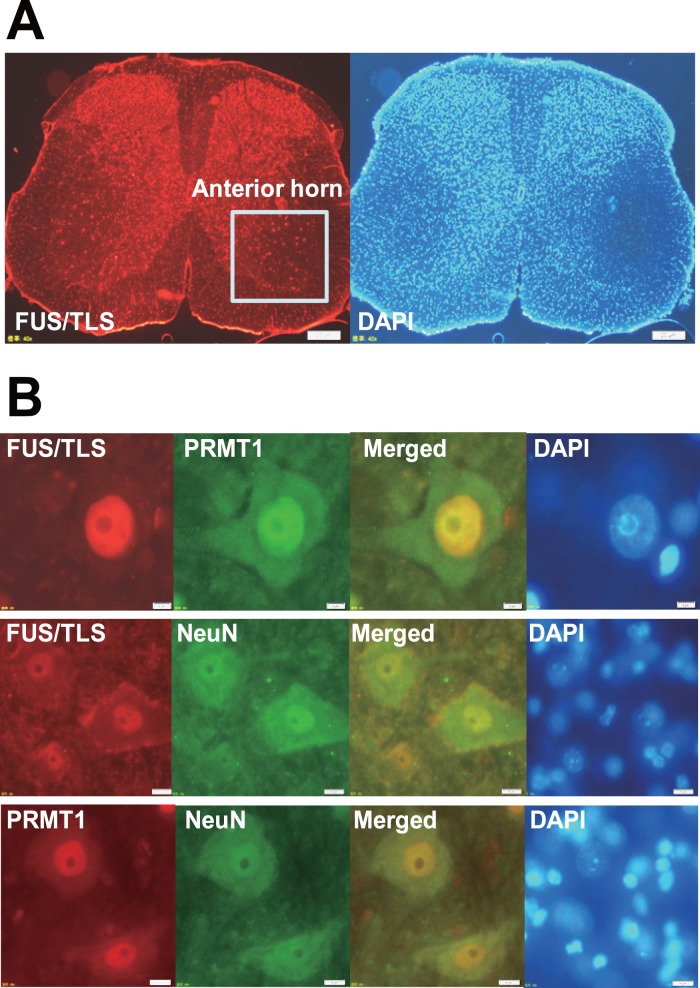
Interaction between FUS/TLS and PRMT1 in spinal cord motor neurons. (A) Mouse lumbar spinal cord sections were stained with anti-FUS/TLS antibody, and followed by the incubation with Alexa568 conjugated secondary antibody (Invitrogen). Sections were then stained with DAPI. Images were obtained using fluorescence microscopy. Square indicates the anterior horn of spinal cord section. Bars, 200 µm. (B) Mouse lumbar spinal cord sections were co-stained with indicated antibodies, followed by the incubation with Alexa568 and Alexa488 conjugated secondary antibody (Invitrogen). Sections were then stained with DAPI. Images were obtained using fluorescence microscopy. Bars, 20 µm.

Next to examine whether FUS/TLS and PRMT1 are co-localized in the neurons of CNS, we performed immunohistochemistry on the sections of mouse brain cortex (Fig. 2B and 2C). The immunoreactivities for FUS/TLS and PRMT1 were co-localized in cortex neurons (Fig. 2B, left panels). The immunoreactivities for FUS/TLS or PRMT1 were co-localized with those for neuronal marker neurofilament or NeuN respectively in brain sections, indicating both proteins are expressed in neurons (Fig. 2B, middle and right panels). At a higher magnification, the immunoreactivities for FUS/TLS and PRMT1 were co-localized primarily in the nuclei of neurons in brain sections (Fig. 2C). To further investigate whether FUS/TLS and PRMT1 are co-localized in motor neurons, we then performed immunohistochemistry on mouse lumber spinal cord sections (Fig. 3A). The immunoreactivities for FUS/TLS and PRMT1 were co-localized primarily in the nucleus of motor neuron, which shows the typical appearance of multipolar neuronal cell bodies with large nucleus (15–20 µm in a diameter) in the anterior horn of spinal cord (Fig. 3B). In addition, the cells positive for FUS/TLS or PRMT1 were co-stained with antibody against NeuN, indicating these are in fact neuronal cells.

### FUS/TLS could be Methylated by PRMT1 *in vitro* and *in vivo*

Since PRMT1 is a major arginine methyltransferase modulating the functions of substrates through methylation [21], we then examine whether FUS/TLS is methylated by PRMT1 using anti-Dimethyl Arginine (DMA) antibody or Anti-dimethyl-Arginine Antibody, asymmetric (ASYM24), which can detect asymmetric dimethyl arginines in the substrates. FUS/TLS contains over 20 arginine glycine (RG)-rich sequences that are potential sites for PRMTs-mediated arginine methylation [37].

**Figure 4:**
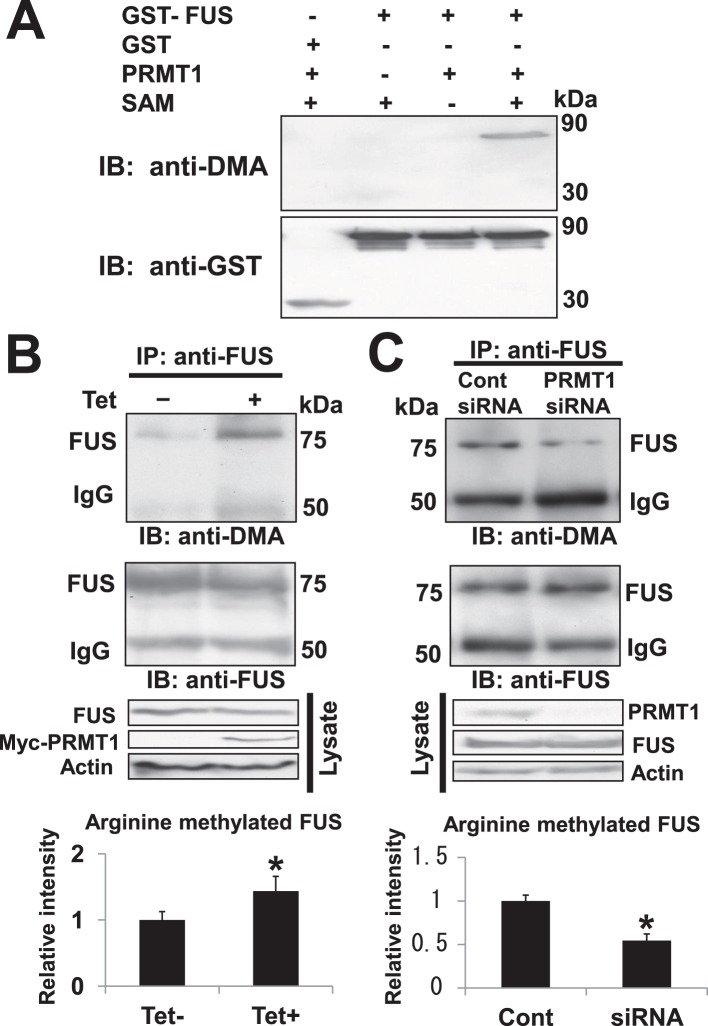
*in vitro* and *in vivo* methylation assay. (A) *in vitro* methylation assay. Bacterial expressed GST-FUS or GST alone (control) was added with recombinant MBP-tagged PRMT1 protein in the presence or absence of S-adenosylmethionine (SAM). The methylation levels of FUS/TLS were monitored using anti-Dimethyl Arginine (DMA) *antibody* (upper panel). The membrane was reprobed with anti-GST antibody to check the loading volume of GST protein in each lane (lower panel). (B) *In vivo* methylation assay. Tet-on inducible PRMT1 over-expression HEK293 cells, treated with 1 µg/ml tetracycline (Tet+) or mock (Tet−) for 24 h, were lysed and immunoprecipitated with anti-FUS antibody. Then immunoprecipitates were blotted with anti-DMA antibody (upper panel). The membrane was reprobed with anti-FUS/TLS antibody to check the loading volume of FUS/TLS protein in each (middle panel). IgG, Immunoglobulin heavy chain. Western blots were scanned using Canon 8800F photo scanner, and densitometric analysis was performed using NIH Ima Graphs represent means of relative intensities in four experiments, corrected to total FUS bands detected by anti-FUS antibody, and expressed as fold over control (value 1). Error bars represent standard deviation. Student’s *t*-test was preformed to evaluate differences between two groups. ^*^p<0.05 vs Tet-. (C) *in vivo* methylation assay. HEK293 cells were treated with PRMT1-targeted siRNA oligonucleotides (PRMT1 siRNA) or control siRNA (Cont siRNA) in a concentration of 40 nM for 24 h. Then lysates of HEK293 cells were immunoprecipitated with anti-FUS/TLS antibody, and the methylation levels of FUS/TLS were monitored with anti-DMA antibody (upper panel). Then membrane was reprobed with anti-FUS antibody to check the loading volume FUS/TLS protein in each lane (middle panel). IgG, Immunoglobulin heavy chain. Western blots were scanned using Canon 8800F photo scanner, and densitometric analysis was performed using NIH ImageJ. Graphs represent means of relative intensities in four experiments, corrected to total FUS bands detected by anti-FUS antibody, and expressed as fold over control (value 1). Error bars represent standard deviation. Student’s *t*-test was preformed to evaluate differences between two groups. ^*^p<0.05 vs Cont siRNA.

We first performed *in vitro* methylation assay using bacterially expressed recombinant MBP-fused PRMT1 and GST-fused FUS/TLS (GST-FUS) or GST alone (control). GST-FUS or GST alone was incubated in the reaction buffer containing PRMT1 in the presence or absence of S-adenosyl methionine (SAM) as the donor of methyl group. After incubation, the samples were resolved on SDS-PAGE, and the methylation status was monitored by the immunoblot with anti-DMA *antibody (* *Fig. 4A*, *upper panel) or ASYM24 (Fig. S2A, upper panel)*. The signals for methylated GST-FUS were observed in the presence of PRMT1 and SAM (Fig. 4A, upper panel, line 4 or Fig. S2A, upper panel, line 4), which was abolished in the absence of PRMT1 or SAM (Fig. 4A, upper panel, line 2–3 or *Fig. S2A, upper panel, line 2–3*). There were no specific signals for GST alone with anti-DMA antibody or *ASYM24* (Fig. 4A, upper panel, line 1 or *Fig. S2A, upper panel, line 1*). The signals for GST-FUS or GST alone protein were comparable in each lane on the membrane reprobed with anti-GST antibody (Fig. 4A, lower panel or Fig. S2A, lower panel), indicating that FUS/TLS could be methylated directly by PRMT1 *in vitro*.

We next examined whether FUS/TLS protein is methylated by PRMT1 *in vivo*. Tet-on inducible over-expressing line was established in HEK293 cell, which conditionally over-expresses Myc-tagged PRMT1 (Myc-PRMT1) in the presence of tetracycline (1 µg/ml). After the treatment with mock or tetracycline (Tet) for 24 hours, cell lysates were immunoprecipitated with anti-FUS/TLS antibody and subjected to the immunoblot with anti-DMA antibody or *ASYM24* to monitor the methylation status. The signals for methylated FUS/TLS were slightly increased in the presence of tetracycline (Fig. 4B, upper panel or Fig. S2B, upper panel) as compared with the control, while the amount of total FUS/TLS protein in the immunoprecipitates was comparable in each lane on the membrane reprobed with anti-FUS antibody (Fig. 4B, middle panel or Fig. S2B, lower panel). To test the specificity of PRMT1-mediated methylation, we performed *in vivo* methylation assay after siRNA-mediated knockdown of PRMT1 in HEK293 cells. We confirmed the expression levels of PRMT1 protein were decreased by PRMT1 siRNA (Fig. 4C, bottom panels) in Western blot analysis, while bands for FUS protein immunoprecipitated by anti-FUS antibody were comparable in each lane (Fig. 4C, middle panel). The signals for arginine methylation in FUS/TLS were slightly decreased by PRMT1 siRNA compared with those for control siRNA (Fig. 4C, upper panel).

### Effect of PRMT1-mediated Arginine Methylation on the Subcellular Localization of FUS/TLS

**Figure 5:**
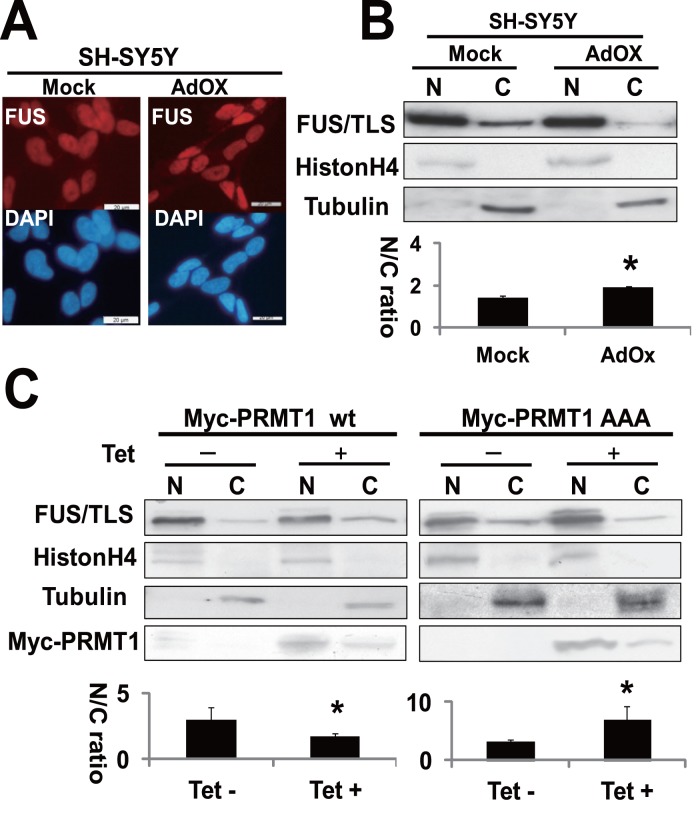
Effect of arginine methylation on the subcellular localization. (A) SH-SY5Y, treated with 20 µM AdOx or Mock for 24 hours, were stained with anti-FUS/TLS antibody, and followed by the incubation with Alexa568 conjugated secondary antibody. Then samples were stained with DAPI, and observed under fluorescence microscopy. Bars, 20 µM. (B) SH-SY5Y cells were treated with 20 µM AdOx for 24 hours, and processed for cell fractionation assays. An aliquot (20 µg) of each extract was separated on 10% SDS-PAGE and subjected to the immunoblots with indicated antibodies. “N” and “C” means nuclear and cytoplasmic fraction respectively. “N/C ratio” means nucleus-cytoplasm ratio calculated as relative intensities in the nucleus/those in the cytoplasm, expressed as mean ±S.D. of 4 separate experiments. ^*^ p<0.05 vs Mock. (C) Tet-on inducible HEK293 cells, treated with 1 µg/ml tetracycline (Tet+) or mock (Tet−) for 24 hours, were processed for cell fractionation assays. An aliquot (20 µg) of each extract was then analyzed by 10% SDS-PAGE and subjected to the immunoblots with indicated antibodies. “N/C ratio” means nucleus-cytoplasm ratio calculated as relative intensities in the nucleus/those in the cytoplasm, expressed as mean ±S.D. of 4 separate experiments. ^*^ p<0.05 vs Tet -.

To examine the effect of methylation status on the subcellular localization of FUS/TLS, we performed immunofluorescence and cell fractionation assays in SH-SY5Y cells. Although the treatment with a general methytransferase inhibitor (AdOx) for 24 hours appeared to have no effect on the subcellular localization of FUS/TLS by immunofluorescence assay (Fig. 5A), cell fractionation assays indicated that AdOx treatment slightly increases the nucleus-cytoplasmic (N/C) ratio of FUS/TLS (or increases nuclear localization) compared with mock treatment (Fig. 5B). We then performed cell fractionation assays using Tet-on inducible Myc-PRMT1 over-expression line of HEK293. Previous report showed that inactive mutant PRMT1 (designated as PRMT1 AAA), in which the enzyme active site is mutated from _63_VLD_65_ to _63_AAA_65_, inhibits the methylation of substrates including hnRNP A2 [38]. Over-expression of Myc-PRMT1 wild type (wt) slightly decreased the N/C ratio of FUS/TLS (or increased the cytosolic localization), while that of PRMT1 AAA increased the ratio (or increased the nuclear localization) (Fig. 5C).

### FUS dC is Partially Co-localized with PRMT1 in the Cytoplasmic Inclusions

**Figure 6:**
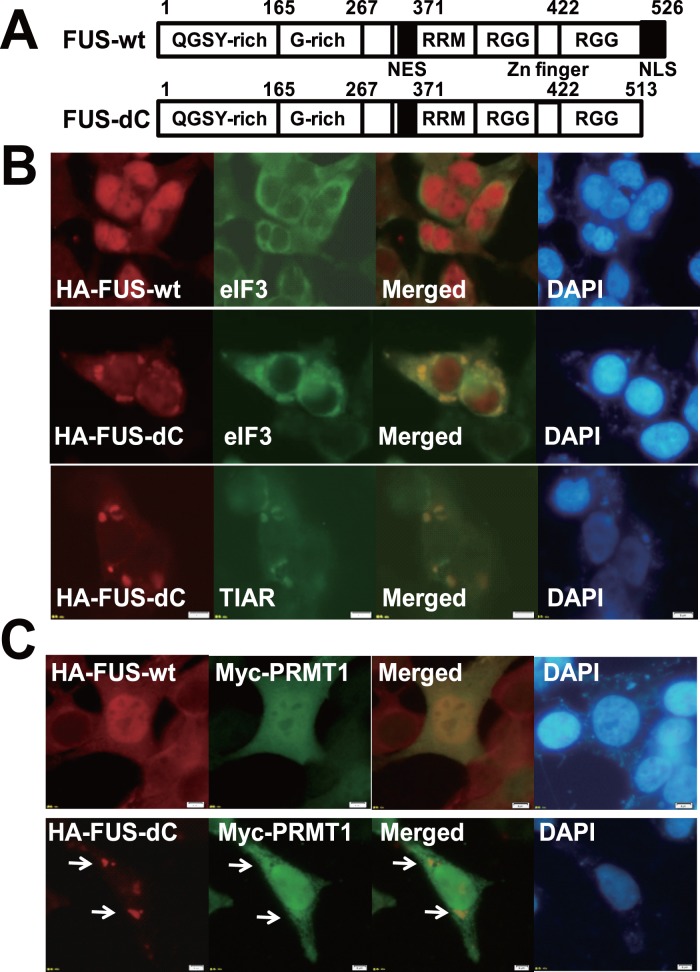
FUS-dC is partially co-localized with PRMT1 in SGs in HEK293 cells. (A) Diagrams for FUS/TLS full length (FUS-wt) and the C-terminal deletion mutant (FUS-dC). QGSY-rich, Gln-Gly-Ser-Tyr-rich region; G-rich, Gly-rich region; RRM, RNA recognition motif; RGG, Arg-Gly-Gly repeats; NLS, nuclear localization signal. (B) HEK293 cells were transiently transfected with plasmid expressing HA-tagged FUS-wt (HA-FUS-wt) or FUS-dC (HA-FUS-dC), co-stained with anti-HA and anti-eIF3 or TIAR antibody, and followed by the incubation with fluorescence conjugated secondary antibody. Samples were then stained with DAPI. Images were obtained using fluorescence microscopy. Bars, 20 µm. (C) HEK293 cells were co-transfected with plasmids expressing HA-FUS-wt (upper panels) or HA-FUS-dC (lower panels) and Myc-tagged PRMT1, then co-stained with anti-HA and anti-Myc antibody and followed by the incubation with fluorescence conjugated secondary antibody. Samples were then stained with DAPI. Images were obtained using fluorescence microscopy. Bars, 20 µm. Arrows indicate the co-localizations.

*FUS/TLS* encodes 526 amino acid protein, characterized by N-terminal QGSY region, G-rich domain, RRM (RNA recognition motif), two RGG (Arg-Gly-Gly repeats), and C-terminal non-classical NLS (514-526 aa) [8], [14] (Fig. 6A). The vast majority of mutations in FUS/TLS-related familial ALS cases were identified in C-terminal NLS, which result in the retention and the inclusion of FUS/TLS in the cytoplasm [2], [3], [5], [8], [14], and FUS/TLS as well as TDP-43 is recruited to SGs under stress conditions such as arsenite, heat shock and hypoxia [28], [29], [30]. Based on these results, we generated a deletion mutant (1-513aa, designated as FUS-dC) that lacks C-terminal non-classical NLS as in Fig.6A. After HEK293 cells were transiently transfected with plasmid encoding HA-tagged FUS wild type (HA-FUS-wt) or FUS-dC (HA-FUS-dC) and Myc-tagged PRMT1 (Myc-PRMT1), immunofluorescence assays were performed (Fig. 6B, 6C). HA-FUS-wt was localized primarily in the nucleus (Fig. 6B, upper panels), however the over-expression of HA-FUS-dC results in the diffused localization pattern with cytoplasmic inclusions (Fig. 6B, middle and bottom panels) like ALS-linked FUS mutants [2], [3], [5], [8], [14]. The immunoreactivities for HA-FUS-dC-positive inclusions were mostly co-localized with those for SGs marker eIF3η and TIA-1-related protein (TIAR) (Fig. 6B, middle and bottom panels). HA-FUS-wt was co-localized with Myc-PRMT1 primarily in the nucleus (Fig. 6C, upper panels), however HA-FUS-dC was co-localized with Myc-PRMT1 partially in the cytoplasmic inclusions (Fig. 6C, lower panels).

### FUS is Partially Co-localized with PRMT1 in the Cytoplasmic Inclusions Under Oxidative Stress by Arsenite

**Figure 7:**
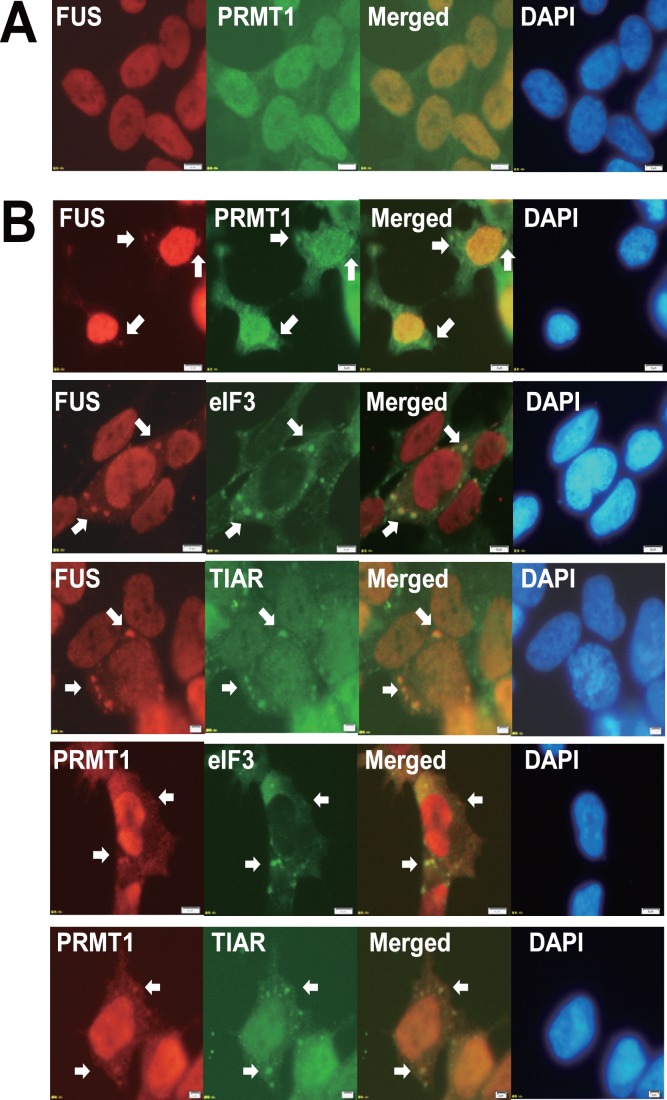
FUS/TLS and PRMT1 are partially co-localized in SGs in SH-SY5Y cells. SH-SY5Y cells were exposed to mock (A) or 0.5 mM arsenite (B) for 30 minutes, then co-stained with indicated antibodies and followed by the incubation with fluorescence conjugated secondary antibody. Samples were then stained with DAPI. Images were obtained using fluorescence microscopy. Bars, 20 µm. Arrows indicate the co-localizations.

We examined whether endogenous FUS/TLS and PRMT1 are co-localized in the cytoplasmic inclusions under stress condition using SH-SY5Y cells. Although co-localized primarily in the nucleus in normal condition (Fig. 7A), FUS/TLS and PRMT1 were partially recruited to the cytoplasmic inclusions when exposed to 0.5 mM arsenite for 30 minutes (Fig. 7B, top panels). The immunoreactivities for cytoplasmic inclusions of FUS/TLS and PRMT1 were partially merged with those for SGs marker eIF3η and TIAR under oxidative stress by arsenite (Fig. 7B, lower panels).

### Conditional Over-expression of PRMT1 wt Reduced FUS-dC-related SGs Formation and Detergent-insoluble FUS-dC Proteins

**Figure 8:**
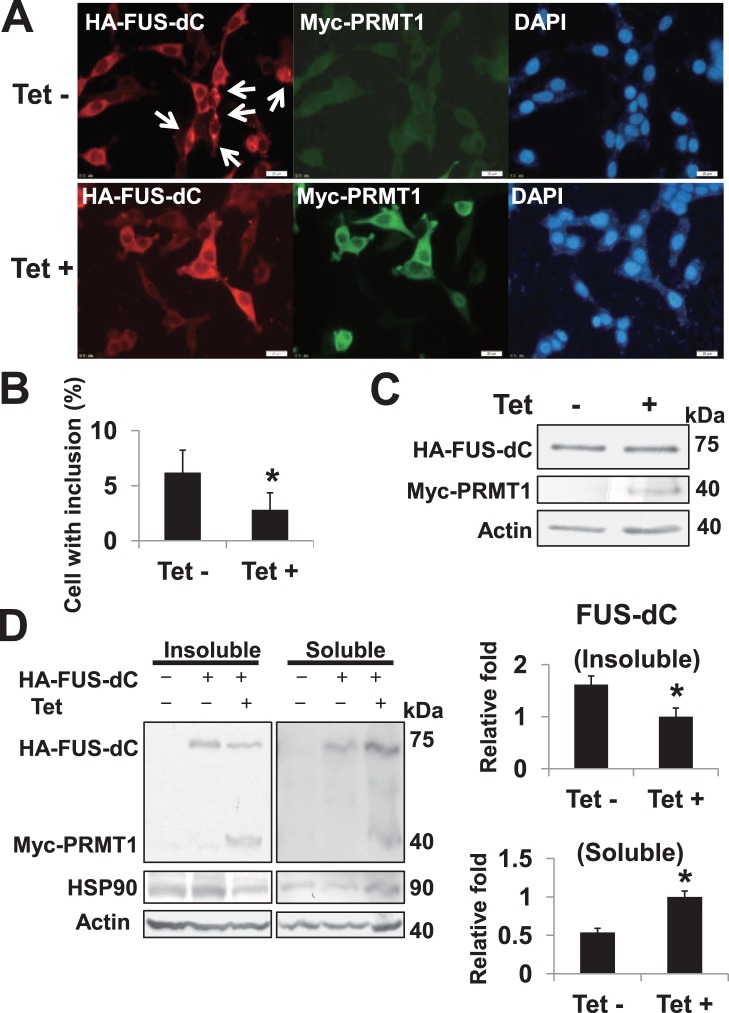
Conditional over-expression of PRMT1 reduces FUS-dC-related aggregates. (A) Tet-on inducible Myc-PRMT1 wt over-expression HEK293 cells, transiently transfected with plasmid encoding HA-tagged FUS-dC, were simultaneously treated with 1 µg/ml tetracycline (Tet+) or mock (Tet−) for 24 hours. Then samples were fixed, co-stained with anti-HA and anti-Myc antibody and followed by the incubation with Alexa488 and Alexa568 conjugated secondary antibody. Samples were then stained with DAPI. Images were obtained using fluorescence microscopy. Bars, 20 µm. Arrows indicate cells harboring HA-FUS-dC-mediated inclusions. (B) The percentage (%) of cells, harboring FUS-dC-mediated inclusions in the presence (Tet +) or absence (Tet −) of tetracycline for 24 hours, was expressed as mean ± S.D. of 4 separate experiments. Cells with at least one visible inclusion were counted as positive. Four dishes were used per experimental group, with at least 100 cells in four random fields being counted on each trial. ^*^ p<0.05 vs Tet- (C) Western blot analysis. Tet-on inducible Myc-PRMT1 wt over-expression HEK293 cells, transiently transfected with plasmid encoding HA-FUS-dC, were treated with 1 µg/ml tetracycline (Tet+) or mock (Tet−) for 24 hours. Then samples were lysed and subjected to Western blot analysis with indicated antibodies. (D) Triton X-100-insoluble fraction assay. Tet-on inducible Myc-PRMT1 wt over-expression HEK293 cells, transiently transfected with plasmid encoding HA- FUS-dC or empty plasmid, were treated with 1 µg/ml tetracycline (Tet+) or mock (Tet−) for 48 hours. Then detergent-soluble (Soluble) and detergent-insoluble (Insoluble) proteins were extracted. Same amounts (30 µg) of soluble and insoluble proteins were loaded in each immunoblot and probed with the indicated antibodies. Immunoreactivity was normalized to the amount of actin. Relative intensities of HA-FUS-dC bands are expressed as mean ±S.D. of 4 separate experiments (right graphs). ^*^p<0.05 vs Tet-.

To investigate the effect of PRMT1-mediated arginine methylation on FUS-dC-related SGs formation, we tested whether the over-expression of PRMT1 alters FUS-dC-mediated inclusions using tet-on inducible Myc-PRMT1 wt over-expression HEK293 line. At 24 hours after the transient transfection of plasmid encoding HA-tagged FUS-dC (HA-FUS-dC), we performed immunofluorescence assays in the presence or absence of tetracycline. FUS-dC-related inclusions were slightly reduced in the presence of tetracycline (Fig. 8A and 8B). Since PRMT1-mediated arginine methylation is implicated in the multiple functions of substrates, including chromatin structure, signal transduction, transcriptional regulation, and RNA metabolism [21], we performed Western blot analysis to rule out the possibility that expression levels of FUS-dC were altered by the over-expression of Myc-PRMT1. The expression levels of HA-FUS-dC protein were comparable in the presence or absence of tetracycline (Fig. 8C). The progressive accumulation of Triton-insoluble mutant SOD1 protein is observed in the spinal cord of familial ALS model G93A SOD1 mice [35]. To capture FUS-dC-related inclusions in *in vitro* experimental system, we employed the triton-solubility experiment as our experimental model and examined the effect of PRMT1 wt over-expression on the detergent-insoluble FUS-dC protein in HEK293 cells. At 48 hours after the transient transfection of plasmid encoding HA-FUS-dC, we performed Western blot analysis using Triton-insoluble fraction (Insoluble) and soluble fraction (Soluble) in tet-on inducible Myc-PRMT1 wt over-expression HEK293 line (Fig. 8D). The detergent-insoluble FUS-dC protein was reduced in the presence of tetracycline (Tet+) compared with the control (Tet−), while soluble FUS-dC is induced in the presence of tetracycline (Tet+) compared with the control (Tet−) indicating PRMT1 wt over-expression could reduce the detergent-insoluble FUS-dC protein. Interestingly molecular chaperon HSP90 is enriched in detergent-insoluble fraction in the absence of tetracycline. Tetracycline and its analogs are known to possess an anti-aggregation activity [39], [40] and might explain the decrease in detergent insoluble fraction of FUS-dC independently of arginine methylation. To exclude this possibility, we examined the effect of Tet treatment on FUS-dC localization and aggregation. First we performed the immunocytochemisty to examine FUS-dC aggregates in the presence or absence of Tet (Fig. S3A) and found the ratio of cells harboring inclusion was comparable between two conditions (Fig. S3A, *p* = 0.47). Next we investigated the effect of Tet treatment on the subcellular localization of FUS-dC and found no significant effect of tetracycline (Fig. S3B, *p* = 0.75). Then we investigated the effect of Tet treatment on FUS-dC in Triton-insoluble or soluble fraction and found that Tet treatment had no significant effect (Fig. S3C, Insoluble, *p* = 0.70; Soluble, *p* = 0.37).

## Discussion

The present study showed that PRMT1 is one of binding partners for FUS/TLS and FUS/TLS could be methylated by PRMT1 *in vitro* and *in vivo*. FUS/TLS and PRMT1 were co-localized primarily in the nucleus of HEK293, SH-SY5Y, and mouse spinal cord motor neuron. Inhibition of PRMT1-mediated methylation of FUS/TLS by a general methyltransferase inhibitor or the over-expression of PRMT1 inactive mutant slightly increased the N/C ratio (or increased the nuclear localization). Conversely over-expression of PRMT1 wild type reduced the ratio (or increased the cytosolic localization), indicating arginine methylation levels could regulate the nucleo-cytoplasmic shuttling. Based on our results, higher levels of arginine methylation could enhance the nuclear export of FUS/TLS or its retention in the cytoplasm. FUS-dC lacking C-terminal NLS formed cytoplasmic inclusions like ALS-linked FUS mutants, which were partially co-localized with PRMT1. Under oxidative stress by arsenite, FUS/TLS and PRMT1 were partially recruited to the cytoplasmic inclusions that were stained with antibody against SGs marker TIAR or eIF3η in SH-SY5Y cells. In addition, conditional over-expression of PRMT1 wt reduced FUS-dC-mediated SGs formation and detergent-insoluble aggregates in HEK293. These findings suggest that PRMT1-mediated arginine methylation could be implicated in the nucleo-cytoplasmic shuttling of FUS/TLS and in the SGs formation and detergent-insoluble aggregates of FUS-dC.

During we are preparing for this paper, Tradewell *et al.* presented that PRMT1 is involved in the nuclear-cytoplasmic localization and toxicity of ALS-linked FUS mutants [41]. Inhibition of PRMT1 by gene knockout or siRNA knockdown reduced the cytoplasmic localization and SGs formation of ALS-linked FUS mutants in mouse embryonic fibroblast and HEK293 cells. Interestingly shRNA-mediated PRMT1 knockdown concomitant with the expression of FUS increased the cytoplasmic localization of ALS-linked FUS mutants, while the pretreatment with a global methyltransferase inhibitor reduced that in primary motor neuron. Most of their results related to the subcellular localization of FUS/TLS are consistent with ours. Since arginine methylation is not a static post-translational modification and there could be redundancy between PRMT family members [21], the methylation status of FUS/TLS might depend on the experimental systems such as gene knockout, siRNA-mediated knockdown, or a general methyltransferase. In fact, the interaction between PRMT8 and FUS/TLS was reported [42], suggesting possible methylation of FUS/TLS by other type I PRMT family members including PRMT2, 3, 4, 6, and 8. In addition, AdOx treatment could only block the methylation of newly synthesized proteins and most cellular methylation is not changed until it is turned over [43], suggesting some fraction of FUS/TLS proteins might be methylated regardless of AdOx treatment. Therefore it appears to be very complicated to understand the effects of arginine methylation on FUS/TLS’s functions in these experimental systems.

During we are preparing for this paper, Dormann *et al.* reported that arginine methylation next to C-terminal NLS is involved in the interaction between FUS/TLS and Transportin [44], which showed that arginine methylation in the domain reduces the binding of Transportin to FUS/TLS. Based on our results and the paper, it is possible that over-expression of PRMT1 wt increases the cytosolic localization by reducing the nuclear import and conversely that of PRMT1 dominant negative form or AdOx treatment increases nuclear localization of FUS/TLS by promoting the nuclear import.

Additionally it is reported that human PRMT1 pre-mRNA is alternatively spliced to yield three variants with distinct N-terminal sequences and these PRMT1 splicing variants localize differently [36]. The subcellular localization of PRMT1 variant 3 used in the present study is both in the nucleus and the cytoplasm as in the Fig.6C, while that of PRMT1 variant 1 or variant 2 is predominantly in the nucleus or in the cytoplasm respectively [36]. The antibody against PRMT1 used in the present study can detect all three variants of PRMT1. As shown in Fig.1D, Fig. 2B, and Fig.3B, immunoreactivities for PRMT1 by this antibody were observed predominantly in the nucleus, suggesting that PRMT1 variant 1 is most abundantly expressed in HEK293, SH-SY5Y, and the neurons of mouse brain or spinal cord.

Numerous papers have already reported that FUS/TLS is a possible substrate for arginine methyltransferase [37], [38], [39], [45], [46]. Du *et al*. reported that FUS/TLS has over 20 RG-repeats, a sequence that is frequently methylated by protein arginine methyltransferases, and at least four RG-repeats are constantly dimethylated [37]. Thus far it remains obscure which RG-repeats in FUS/TLS are precisely PRMT1-mediated arginine methylation sites. In general, arginine methylation of heterogeneous ribonucleoproteins (hnRNPs) has numerous effects on biological properties. Arginine methylation is implicated in nucleo-cytoplasmic shuttling of hnRNPs and also alters the RNA-binding properties and protein-protein interactions of RG-rich proteins, including hnRNP A1 [47], hnRNP A2 [48], and SAM68 [49]. FMRP, the fragile X mental retardation protein, is also methylated by PRMT, affecting its protein-protein interactions, polyribosome association and regulating the translation of its target mRNAs [50], [51]. Especially arginine methylation is required for nuclear export of hnRNPs, including Npl3p and Hrp1p in the yeast and hnRNP A2 in mammalian cells [48], [52] These reports suggest the biological effects of arginine methylation on FUS/TLS by PRMT1, including nucleo-cytoplasmic shuttling, RNA-binding properties, protein-protein interaction, polyribosome association and translational regulation of its target mRNAs. In addition, the deletion of two major yeast arginine methyltransferases did not regulate the localization and toxicity of FUS/TLS in yeast model [53], raising the possibility that arginine methylation of FUS/TLS might not be essential for subcellular localization and toxicity even in mammalian cells.Recently N-terminal region of FUS/TLS is reported to be a prion-related domain that is enriched in uncharged polar amino acids [especially asparagine (N), glutamine (Q), and tyrosine(Y)] [54], [55], [56] and required for the formation of FUS-mediated aggregates with additional RGG domain in yeast and mammalian cell [53]. Furthermore only the prion-related domain of TIA-1, an RNA binding protein required for SGs formation under stress condition, forms aggregates that are recruited to SGs 57. Therefore it is possible the prion-related domain of FUS/TLS could mediate the aggregates of FUS/TLS proteins in neuron or glia leading to the pathogenesis of ALS and FTLD, once wild type FUS/TLS protein is recruited to SGs under physiological stress conditions. This could be consistent with our result in which PRMT1 over-expression reduced not only FUS-dC-related SGs formation but also detergent insoluble FUS-dC aggregates in HEK293 cells.

What is the mechanism in which PRMT1 over-expression reduces FUS-dC-related SGs formation and detergent-insoluble FUS-dC aggregates in the present study? It appeared to be a discrepancy that the arginine methylation induces the cytosol-existing FUS/TLS but reduces cytosolic FUS-dC-related SGs formation. It is proposed that protein methylation plays some role in stress granule recruitment and remodeling rather than in its assembly or disassembly [58], [59]. One simple explanation is that the addition of methyl groups to arginine side chain has some inhibitory effect on the prion-related domain of FUS/TLS, reducing the formation of FUS-dC-related SGs and aggregates. Fig. 8A (middle, lower) shows morphologically that some amount of HA-FUS-dC is co-localized diffusedly with Myc-PRMT1 in the cytoplasm. In addition, Myc-PRMT1 protein was detected in the detergent-insoluble fraction with HA-FUS-dC (Fig. 8D), raising the possibility that PRMT1 binds to FUS-dC preventing its aggregation like chaperon independently of arginine methylation. Since PRMT1 variant 3 localizes more in the cytoplasm compared to PRMT1 variant 1, this could confer some effects. Another possibility is the indirect effects of arginine methylation on other proteins, involved in the formation of SGs and detergent-insoluble aggregates mediated by FUS-dC. Molecular chaperon could be one of prominent candidates for this. To further examine the interaction between the arginine methylation and the FUS/TLS-related SGs formation or aggregates could hopefully contribute to the development of a novel therapy for incurable ALS and FTLD in the future.

## Supporting Information

Figure S1 **The interaction between FUS and PRMT1 in the presence of nuclease.** Co-immunoprecipitation assay between endogenous FUS/TLS and PRMT1 in the presence of RNase A and DNase I. The lysates of HEK293 cells were immunoprecipitated with anti-FUS/TLS antibody or control mouse IgG. After FUS/TLS immunoprecipitation, samples were treated with 1 µg/ml RNase A and 10 U/ml DNase 1 or mock at 37°C for 1 h. Then immunoprecipitates were immunoblotted with anti-PRMT1 (upper panel) or anti-FUS/TLS antibody (lower panel). IgG HC, Immunoglobulin heavy chain.
(EPS)

Figure S2 ***In vivo*** **and** ***in vitro*** **methylation assay with ASYM24.** (A) *In vitro* methylation assay with ASYM24. The membrane used in Fig.4A was reprobed with ASYM24 in this assay. Briefly, bacterial expressed GST-FUS or GST alone (control) protein was added with recombinant MBP-tagged PRMT1 protein in the presence or absence of S-adenosylmethionine (SAM). The methylation levels of FUS/TLS were monitored with ASAM24 (upper panel). Arrow indicates methylated GST-FUS protein, and asterisk denotes possible MBP-PRMT1 bands. The membrane was also reprobed with anti-GST antibody to check the loading volume of GST protein in each lane (lower panel). (B) *In vivo* methylation assay with ASYM24. The membrane used in Fig.4B was reprobed with ASYM24 in this assay. Briefly, Tet-on inducible PRMT1 over-expression HEK293 cells, treated with 1 µg/ml tetracycline (Tet+) or mock (Tet−) for 24 h, were lysed and immunoprecipitated with anti-FUS antibody. Then immunoprecipitates were blotted with ASYM 24 (upper panel). The membrane was also reprobed with anti-FUS/TLS antibody to check the loading volume of FUS/TLS protein in each lane (lower panel). IgG, Immunoglobulin heavy chain. Western blots were scanned using Canon 8800F photo scanner, and densitometric analysis was performed using NIH ImageJ. Graphs represent means of relative intensities in four experiments, corrected to total FUS bands detected by anti-FUS antibody, and expressed as fold induction over control (standardized as value 1). Error bars represent standard deviation. Student’s *t*-test was preformed to evaluate differences between two groups.
(EPS)

Figure S3 **Tetracycline has no effect on the interaction between FUS/TLS and PRMT1.** (A) HEK293 cells, transiently transfected with plasmid encoding HA-tagged FUS-dC, were treated with 1 µg/ml tetracycline (Tet+) or mock (Tet−) for 24 hours. Then samples were fixed, stained with anti-HA antibody and followed by the incubation with Alexa568 conjugated secondary antibody. Samples were then stained with DAPI. Images were obtained using fluorescence microscopy. Bars, 20 µm. The percentage (%) of cells, harboring FUS-dC-mediated inclusions in the presence (Tet +) or absence (Tet −) of tetracycline for 24 hours, was expressed as mean ± S.D. of 3 separate experiments. Cells with at least one visible inclusion were counted as positive. Four dishes were used per experimental group, with at least 100 cells in four random fields being counted on each trial. (B) HEK293 cells, treated with 1 µg/ml tetracycline (Tet+) or mock (Tet−) for 24 hours, were processed for cell fractionation assays. An aliquot (20 µg) of each extract was then analyzed by 10% SDS-PAGE and subjected to the immunoblots with indicated antibodies. “N/C ratio” means nucleus-cytoplasm ratio calculated as relative intensities in the nucleus/those in the cytoplasm, expressed as mean ±S.D. of 3 separate experiments. (C) Triton X-100-insoluble fraction assay. HEK293 cells, transiently transfected with plasmid encoding HA- FUS-dC or empty plasmid, were treated with 1 µg/ml tetracycline (Tet+) or mock (Tet−) for 48 hours. Then detergent-soluble (Soluble) and detergent-insoluble (Insoluble) proteins were extracted. Same amounts (30 µg) of soluble and insoluble proteins were loaded in each immunoblot and probed with the indicated antibodies. Immunoreactivity was normalized to the amount of actin. Relative intensities of HA-FUS-dC bands over control (standardized as value 1) were expressed as mean ±S.D. of 3 separate experiments (right graphs).
(EPS)
